# Fatigability and Cardiorespiratory Impairments in Parkinson’s Disease: Potential Non-Motor Barriers to Activity Performance

**DOI:** 10.3390/jfmk5040078

**Published:** 2020-10-31

**Authors:** Andrew E. Pechstein, Jared M. Gollie, Andrew A. Guccione

**Affiliations:** 1 Department of Rehabilitation Science, George Mason University, Fairfax, VA 22030, USA; apechste@masonlive.gmu.edu (A.E.P.); aguccion@gmu.edu (A.A.G.); 2 Research Services, Veterans Affairs Medical Center, Washington, DC 20422, USA; 3 Department of Health, Human Function, and Rehabilitation Sciences, The George Washington University, Washington, DC 20006, USA

**Keywords:** oxygen consumption, perceived fatigability, endurance, walking, cardiorespiratory fitness, aerobic metabolism, autonomic nervous system

## Abstract

Parkinson’s disease (PD) is the second most common neurodegenerative condition after Alzheimer’s disease, affecting an estimated 160 per 100,000 people 65 years of age or older. Fatigue is a debilitating non-motor symptom frequently reported in PD, often manifesting prior to disease diagnosis, persisting over time, and negatively affecting quality of life. Fatigability, on the other hand, is distinct from fatigue and describes the magnitude or rate of change over time in the performance of activity (i.e., performance fatigability) and sensations regulating the integrity of the performer (i.e., perceived fatigability). While fatigability has been relatively understudied in PD as compared to fatigue, it has been hypothesized that the presence of elevated levels of fatigability in PD results from the interactions of homeostatic, psychological, and central factors. Evidence from exercise studies supports the premise that greater disturbances in metabolic homeostasis may underly elevated levels of fatigability in people with PD when engaging in physical activity. Cardiorespiratory impairments constraining oxygen delivery and utilization may contribute to the metabolic alterations and excessive fatigability experienced in individuals with PD. Cardiorespiratory fitness is often reduced in people with PD, likely due to the combined effects of biological aging and impairments specific to the disease. Decreases in oxygen delivery (e.g., reduced cardiac output and impaired blood pressure responses) and oxygen utilization (e.g., reduced skeletal muscle oxidative capacity) compromise skeletal muscle respiration, forcing increased reliance on anaerobic metabolism. Thus, the assessment of fatigability in people with PD may provide valuable information regarding the functional status of people with PD not obtained with measures of fatigue. Moreover, interventions that target cardiorespiratory fitness may improve fatigability, movement performance, and health outcomes in this patient population.

## 1. Introduction

Parkinson’s disease (PD) is the second most common neurodegenerative condition after Alzheimer’s disease, affecting an estimated 160 per 100,000 people 65 years of age or older [1]. With advancing age considered to be the greatest risk factor for developing PD, the projected expansion of the older adult population portends a sharp increase in the burden of PD in coming years [2]. PD is characterized by both motor and non-motor symptoms with the cause(s) including the interplay of genetic and environmental factors [3,4]. Motor dysfunction is thought to occur due to dopamine deficiency within the basal ganglia as a result of the death of dopaminergic neurons in the substantia nigra pars compacta [3]. However, other key dopaminergic areas of the brain may also contribute, in part, to some of the non-motor symptoms identified [4]. Furthermore, non-motor symptoms may present themselves earlier in the disease process compared to motor symptoms, which become more prominent during later stages of disease progression, and therefore may provide a more valuable prognostic indicator for the identification of individuals at risk for PD.

Fatigue is a debilitating non-motor symptom frequently reported in PD [3,4,5,6]. While fatigue has garnered much scientific and clinical interest as a symptom in PD, recent efforts have been made to distinguish the symptom of fatigue from fatigability [6]. It has been proposed that by differentiating these two constructs, unique health and functional characteristics can be ascertained [6]. Fatigability is distinct from fatigue in that it not only describes elements of human performance but also provides a framework for identifying impairments that ultimately may drive activity limitation [6]. Importantly, fatigability encompasses systems associated with perceptions of effort and sensations not associated with the symptom of fatigue, in addition to processes required for excitation–contraction coupling [6]. Greater susceptibility to fatigability may also underscore findings of increased physical inactivity in PD [7]. Currently, the concept of fatigability and its relationship to fatigue are not fully appreciated in the PD literature. This may not only hinder a more precise and robust understanding of fatigability in PD, but also may be a missed opportunity to understand a critical dimension of activity limitation in members of this patient population. Therefore, the purpose of this review is to (1) highlight key differences between the constructs of fatigue and fatigability, (2) outline evidence of elevated fatigability in PD, (3) describe fatigability during whole-body activity from a bioenergetic perspective, and (4) identify potential cardiorespiratory impairments contributing to fatigability in PD.

## 2. Fatigue versus Fatigability

### 2.1. Fatigue as a Symptom

Fatigue is a symptom defined as a subjective lack of physical and/or mental energy that is perceived by the individual to interfere with usual or desired activities commonly assessed using self-report questionnaires [8]. In PD specifically, fatigue has been characterized as a sense of exhaustion that is unexplained by drug effects or other medical or psychiatric disorders [5,6]. Fatigue is commonly reported in people with PD, often manifesting prior to disease diagnosis, persisting over time, and negatively affecting quality of life, even when controlling for other symptoms such as depression, motor symptom severity, and sleep quality [9,10,11,12,13,14,15,16]. While the exact mechanisms causing excessive fatigue in PD are not entirely understood, proposed mechanisms include increased circulating pro-inflammatory cytokines, dysfunction in nigrostriatal and extrastriatal dopaminergic pathways, involvement of non-dopaminergic pathways, executive/prefrontal pathology, involvement of the autonomic nervous system, and low-grade systemic hypotension [6,17]. People with PD frequently identify fatigue as their most (or among their most) debilitating symptom(s) [9,18]. Importantly, increased fatigue has been shown to be associated with decreased physical activity in PD, a relevant factor given that exercise and a physically active lifestyle may slow or halt the progression of the disease [19,20,21,22,23]. However, a disassociation between objective measures of performance fatigability and the symptom fatigue have been reported in PD, suggesting that the underlying mechanisms contributing to fatigability and fatigue likely differ [24].

### 2.2. Fatigability: An Activity-Induced Construct

Fatigability is distinct from fatigue in that it describes the magnitude or rate of change over time in the performance of activity (i.e., performance fatigability) and sensations regulating the integrity of the performer (i.e., perceived fatigability) [25]. Thus, fatigability is a direct measure of human performance that results from engaging in any physical activity. Given its link to physical activity, fatigability has been proposed as a prognostic indicator of phenotypic aging and physical resilience [26,27]. Therefore, individuals who express greater levels of fatigability, performance or perceived, are likely to be limited in their ability to sustain physically demanding activities. While fatigability has been relatively understudied in PD as compared to fatigue, it has been hypothesized that the presence of elevated levels of fatigability in PD results from the interactions of homeostatic, psychological, and central factors [28]. The central underlying tenant of this premise is that individuals with PD are compromised in their ability to regulate changes in energetic demands during physical activity.

## 3. Evidence of Elevated Fatigability in Parkinson’s Disease

The literature indicates that both performance fatigability and perceived fatigability are often elevated in people with PD. Studies providing insight into fatigability during whole-body activities in people with PD are presented in Table 1 [29,30,31,32,33,34,35,36,37,38,39,40]. When compared to healthy controls, it is consistently reported that people with PD demonstrate reduced distance walked and lower cardiorespiratory fitness [29,30,33,35,38,39]. Multiple reports of reduced performance during less demanding exercise protocols in people with PD compared to healthy controls [31,37,40,41] suggest these individuals are likely predisposed towards increased fatigability during less strenuous activities as well. Elevated perceived fatigability often occurs concomitantly with increased performance fatigability in people with PD. Ratings of perceived exertion have been reported to be higher at peak exercise in those with PD compared to healthy controls despite lower peak work rates [33,35]. Such findings indicate that individuals with PD are at greater risk for experiencing higher levels of perceived effort even when performing activities of lower intensities. Older adults without PD perform activities of daily living closer to their maximal capacities, requiring a greater level of relative effort [42]. Thus, excessive levels of fatigability in PD may be reflective of the inability to meet the bioenergetic demands required to perform a given activity due to the combination of reduced physiological capacity and impaired bioenergetic processes.

## 4. Fatigability from a Bioenergetic Perspective

The cardiorespiratory system, in conjunction with skeletal muscle bioenergetic systems, supports the energy synthesis required to maintain muscle contraction. Cross-sectional and interventional studies have provided evidence of a strong association between cardiorespiratory fitness and fatigability in individuals across a wide range of ages and patient populations [43,44,45,46,47,48,49,50,51]. Biological aging results in declines in cardiorespiratory fitness due to reductions in cardiac output and arteriovenous oxygen difference beginning around the third decade with accelerated decreases occurring around the sixth decade and beyond [45]. Insufficient oxygen delivery is considered the primary factor contributing to activity termination during maximal aerobic activity [52,53]. In contrast, activity termination during prolonged submaximal activity results from factors related to oxygen utilization and/or perceived effort associated with activity performance [52,53,54]. During dynamic muscular activity, fatigability is shown to be strongly associated with increased metabolite accumulation in older adults due to a preferential reliance on anaerobic bioenergetic systems [55]. The rise in metabolite concentrations is implicated as an underlying mechanism contributing to impaired skeletal muscle cross-bridge formation and heightened sensations of activity-induced discomfort [56,57,58,59,60,61].

The signs and symptoms of PD are typically seen as emerging from a primarily “neurological” condition, however, reduced cardiorespiratory fitness may be an underappreciated contributor to elevated fatigability in this population. While studies investigating peak oxygen consumption (VO_2_) between people with PD and controls are not conclusive [29,30], most findings point to lower peak VO_2_ in people with PD. Moreover, the literature widely reports reductions in various submaximal indices of aerobic function and substantial limitations in exercise performance in the PD population [29,30,31,33,35,37,38,39,40,41,62]. Several factors are likely to contribute to low cardiorespiratory fitness in PD including cardiorespiratory alterations due to aging, physical inactivity, and cardiorespiratory impairments specific to the pathophysiology of PD. For example, adults 65 years of age and older comprise the largest portion of the PD population and are thus likely to be impacted by age-related declines in cardiorespiratory fitness [63,64,65,66]. High levels of physical inactivity have been reported in people with PD which were found to be associated with greater disease severity, gait impairment, and greater disability in daily living [7]. In addition to these factors, individuals with PD often require greater energetic costs during activities such as walking [67]. The increased energetic cost combined with lower cardiorespiratory fitness places greater strain on the cardiorespiratory and skeletal muscle bioenergetic systems to meet the energetic requirements for activity. The decreased capacity to support aerobic energy synthesis promoting increased anaerobic metabolic contributions may explain the potential elevations in performance fatigability and perceived fatigability in individuals with PD [28]. The subsequent sections of this review will detail how cardiorespiratory impairments, especially alterations in oxygen delivery and oxygen utilization, are likely to influence fatigability in this patient population.

## 5. Cardiorespiratory Impairments Contributing to Fatigability in Parkinson’s Disease

**Figure 1:**
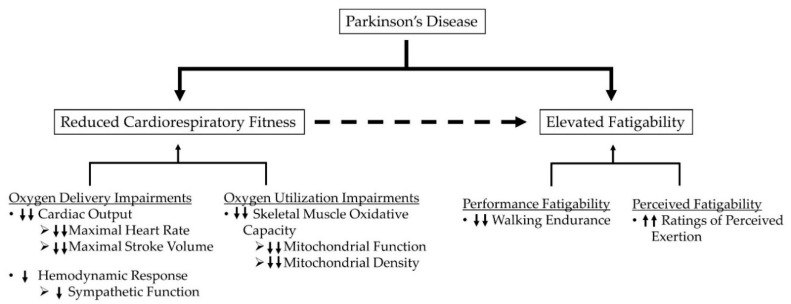
Hypothesized schematic representation of potential relationships between PD, fatigability, and cardiorespiratory impairments. Single arrows represent the impairments independently related to PD, while double arrows represent impairments related to the combination of PD and aging.

To date, factors contributing to performance fatigability and perceived fatigability in PD during various activities have not been fully elucidated. Given that age is a primary risk factor for PD, age-related factors limiting oxygen delivery and utilization during physical activity present themselves as likely candidates contributing to elevations in fatigability in PD. These factors include reductions in cardiac output, redistribution of blood flow away from oxidative fibers, impaired endothelium-mediated vasodilation, and potential reductions in muscle mitochondrial density and function [66,68,69,70,71,72,73,74]. As such, insufficient oxygen delivery and utilization may constrain skeletal muscle respiration in older adults, placing greater reliance on anaerobic bioenergetic systems for energy synthesis. Additionally, insufficient blood flow in older adults may also impair metabolite removal which further promotes metabolite accumulation, thus exacerbating deficits in muscle performance and increasing sensations associated with effort and exertion [60,61,75,76]. Beyond potential age-related constraints, several observations support the notion that factors specific to the pathophysiology of PD further compound age-related alterations in oxygen delivery and utilization. Figure 1 depicts the potential relationships between PD, fatigability, and cardiorespiratory impairments.

### 5.1. Oxygen Delivery

Autopsy investigations [77,78] and studies employing functional imaging or catheterization techniques revealed sympathetic denervation of cardiac muscle in people with PD [77,79,80,81,82,83,84,85,86,87,88,89,90,91,92,93,94,95,96]. The alteration in sympathetic activity is found to occur early in the disease process [97], sometimes even preceding the onset of clinically apparent motor symptoms [98,99], and increases in severity with disease progression [100]. Individuals with PD often fail to reach their age-predicted maximal heart rate (HR) at peak exercise [33,40,101]. Increased fatigability is often reported alongside decreased peak VO_2_ and decreased peak HR in people with PD compared to healthy controls [35,38,39,62]. In addition to aberrant HR responses, cardiac contractility may also be impaired in people with PD, the severity of which likely corresponds to the degree of sympathetic denervation of the heart [41].

Degeneration of key cardiovascular control centers in the brain [100,102,103,104,105,106,107,108] and sympathetic denervation in the thyroid and renal cortex have also been reported in people with PD [81,109,110]. While the collective influence of these pathological alterations on hemodynamics and exercise performance is not completely described, reduced systolic blood (BP) pressure responses to exercise are commonly observed in people with PD [31,33,35,39,111]. For example, it was recently reported that, in comparison to healthy controls, systemic vascular resistance dropped more precipitously upon initiation of exercise in people with PD [40], suggesting that sympathetic control over vascular conductance may be reduced in members of this patient population. Additionally, blunted increases in systolic BP have been associated with increased fatigability [33] and reduced VO_2_ in people with PD compared to healthy controls [35,39]. Factors that are likely relevant to loss of control over arterial pressure during activity in people with PD include baroreflex failure [109], impaired metaboreflex vasoconstrictor responses [112,113,114], decreased muscle sympathetic nerve activity [115,116], reduced norepinephrine release [35,109,117,118], or a confluence of these factors in combination with reduced cardiac performance. Failure to adequately increase systolic BP during exercise, which can occur secondary to either reduced cardiac output or loss of sympathetic control over vascular conductance, may compromise perfusion of active muscle [119,120]. Inadequate muscle perfusion negatively impacts aerobic metabolism [119,121], reduces skeletal muscle contractile performance [122,123,124], and contributes to elevated sensations of activity-induced discomfort. Thus, these data posit limitations in maximal HR, reductions in cardiac contractility, and deleterious alterations in sympathetic control over vascular conductance as factors that may constrain oxygen delivery to working muscles and contribute to increased fatigability in people with PD.

### 5.2. Oxygen Utilization

Cardiorespiratory fitness and fatigability are also affected by the functional state of skeletal muscle and factors that influence oxidative phosphorylation [73]. Although one of the earliest studies of skeletal muscle in PD provided anatomical evidence to suggest that mitochondria were negatively impacted in this patient population [125], data from subsequent studies investigating the possibility of muscle mitochondrial impairment in PD were inconclusive [126,127,128,129,130,131,132,133,134,135,136], perhaps due to methodological differences [137]. More recently, studies have corroborated findings of impaired respiratory capacity in mitochondria [137,138,139]. Although a critique of the evidence of mitochondrial impairments in PD is outside the scope of this review, future research should consider whether compromised oxygen utilization contributes to fatigability in this patient population, especially when engaging in activities reliant on oxidative energy synthesis such as prolonged walking. Structural and functional alterations in the intrinsic dynamics of oxidative phosphorylation may impair aerobic energy synthesis and elevate fatigability [73]. However, the degree to which fatigability and reduced cardiorespiratory fitness in PD are attributable to deficits in oxygen utilization is not well established as these relationships have not been directly assessed in this patient population.

## 6. Clinical Considerations

Increased fatigability in PD may be considered a sign of low exercise capacity, impending frailty, and negative health outcomes, and is therefore important to monitor clinically [26,27]. The more rapid deterioration of performance and greater sense of exertion during activity may discourage individuals with PD from initiating positive behavior changes towards a more physically active lifestyle [140]. Aerobic exercise training has garnered increased attention in PD as a complimentary treatment to pharmacologic options due to its potential positive impact on motor symptoms, cognition, brain health, and disease progression [20,141,142]. Additionally, as noted by a recent systematic review, aerobic training appears to be a viable strategy for improving cardiorespiratory fitness as measured by peak VO_2_, performance fatigability as measured by walking distance, peak work rate, or exercise test duration, and perceived fatigability as measured by ratings of perceived exertion during walking tests [143]. However, results are not uniform and large variation between studies in terms of frequency and intensity of training, program duration, and testing mode (i.e., treadmill vs. bicycle) makes it difficult to determine which elements of the overall program design are most essential for improving cardiorespiratory fitness and fatigability. Moreover, exposure to sufficient exercise intensity and volume may be difficult in people with PD who experience excessive fatigability during physical activity and exercise. Training parameters, therefore, such as intensity, volume, and duration, should be carefully manipulated in an individualized fashion to gradually introduce and increase workloads and maximize tolerability. Patients should always consult with a physician prior to engaging in exercise and may benefit from working with a multidisciplinary team of medical and rehabilitation professionals to improve safety and maximize benefits of training. Future studies should aim to establish the validity and reliability of fatigability measures in the PD population and to determine the effectiveness of exercise interventions targeting cardiorespiratory fitness and fatigability.

## 7. Summary

Fatigue and fatigability, when viewed as distinct constructs and assessed separately, provide unique health and functional information. This article extends current understandings of fatigability in PD by positing disturbances in metabolic homeostasis resulting from cardiorespiratory impairments as a potential explanation for elevated fatigability in this population. The exact contributions of biological aging and the pathophysiological consequences of PD to diminished cardiorespiratory fitness in PD are not completely known, however, this article illuminates several plausible factors likely to impair oxygen delivery and oxygen utilization during physical activity. Future research is needed to determine which fatigability assessments are most appropriate for individuals with PD as well as the responsiveness of fatigability measures to therapeutic interventions.

## Figures and Tables

**Table 1:** Studies characterizing fatigability during whole-body activities in people with Parkinson’s disease (PD).

Author(s), Year	Sample Size (*n*), Age (Years)	Assessment	Results: Performance Fatigability	Results: Perceived Fatigability
Protas et al., 1996 [29]	PD: *n* = 8, 61.4 (6.9) CON: *n* = 7, not reported	Leg cycle ergometer to volitional exhaustion (20 W increase every 2 min). Arm cycle ergometer to volitional exhaustion (10 W increase every 2 min)	Peak power output significantly lower in PD vs. CON during leg and arm cycle ergometer tests.	n/a
Stanley et al., 1999 [30]	PD: *n* = 20, not reported CON: *n* = 23, not reported	Leg cycle ergometer with 20 W increase every 2 min until volitional exhaustion	Peak exercise time significantly lower in PD men vs. CON men. No difference in peak exercise time in PD women vs. CON women.	n/a
Reuter et al., 1999 [31]	PD: *n* = 15, 63 (6.17) CON: *n* = 15, 63.8 (5.38)	Leg cycle ergometer with 4 min stages (25 W, 50 W, 75 W, 100 W, 125 W, 150 W)	% completed each stage (PD vs. CON): (75 W: 100% vs. 100%) (100 W: 80% vs. 100%) (125 W: 47% vs. 80%) (150 W: 47% vs. 60%)	n/a
Canning et al., 2006 [32]	PD: *n* = 16, 65 (6.9) CON: *n* = 22, 66.3 (6.6)	6-Minute Walk Test	Significantly lower total distance walked in PD vs. CON.	Perceived leg fatigue at end of test significantly higher in PD vs. CON. Mean perceived shortness of breath at end of test higher in PD vs. CON.
Werner et al., 2006 [33]	PD: *n* = 16, not reported CON: *n* = 11, 66.1 (9.4)	Treadmill Modified Bruce Protocol	Mean peak work rate lower in PD vs. CON.	RPE significantly higher at peak exercise in PD vs. CON.
Christiansen et al., 2009 [34]	PD: *n* = 90, 64.4 (10.3) CON: *n* = 44, 64.6 (7.3)	Treadmill walking at different speeds (1 mph, 1.5 mph, 2 mph, 2.5 mph, 3 mph, 3.5 mph)	n/a	RPE significantly higher during walking in PD vs. CON (2 mph, 2.5 mph, 3 mph, 3.5 mph).
DiFransisco-Donoghue et al., 2009 [35]	PD: *n* = 14, 67.7 (6.8) CON: *n* = 15, 67.1 (4.4)	Treadmill Modified Bruce Protocol	Peak exercise time significantly lower in PD vs. CON.	Mean RPE higher at peak exercise in PD vs. CON.
Maggioni et al., 2012 [36]	PD: *n* = 14, 67.9 (8.1) CON: *n* = 14, 66.6 (5.3)	5-Minute Walk Test at both “self-selected” and “as fast as possible” speeds	Total distance walked significantly lower in PD vs. CON at “self-selected” and “as fast as possible” speeds.	n/a
Speelman et al., 2012 [37]	PD: *n* = 546, not reported CON: *n* = 29, not reported	Astrand–Rhyming submaximal leg cycle ergometer exercise test	% test completion (PD vs. CON): (46% vs 86%).	n/a
Strano et al., 2016 [38]	PD: *n* = 18, 59.3 (10.5) CON: *n* = 18, not reported	Leg cycle ergometer using individualized ramp protocols designed to promote volitional exhaustion in 6–12 min	Peak power output significantly lower in PD vs. CON.	n/a
Kanegusuku et al., 2016 [39]	PD: *n* = 48, 66 (8) CON: *n* = 20, 64 (9)	Leg cycle ergometer using individualized ramp protocols designed to promote volitional exhaustion in 8–12 min	Workload at RCP significantly lower in PD vs. CON. Mean workload at AT lower in PD vs. CON.	n/a
Roberson et al., 2019 [40]	PD: *n* = 14, 68 (12) CON: *n* = 16, 66 (7)	Leg cycle ergometer using 3 min stages (35 W, 55 W, 75 W, 95 W, 115 W)	% completed each stage (PD vs. CON): (stage 1: 100% vs. 100%) (stage 2: 93% vs. 100%) (stage 3: 71% vs. 81%) (stage 4: 43% vs. 75%) (stage 5: 0% vs. 38%).	Mean RPE higher at peak exercise in PD vs. CON.

Table 1 Search Strategy: relevant studies were retrieved through PubMed and Google Scholar databases using search terms “Parkinson”, “exercise”, “walking”, cardiopulmonary”, “cardiovascular”, and “treadmill” with no publication date restrictions applied. The reference lists of identified articles were also searched. Only cross-sectional studies with comparisons to a healthy referent group were included. Age is presented as mean (SD). Abbreviations: PD—Parkinson’s disease; CON—healthy referent subjects; W—watts; RPE—ratings of perceived exertion; RCP—respiratory compensation point; AT—anaerobic threshold; n/a—not applicable.
